# Targeting the *Mycobacterium ulcerans* cytochrome *bc*_*1*_:*aa*_*3*_ for the treatment of Buruli ulcer

**DOI:** 10.1038/s41467-018-07804-8

**Published:** 2018-12-18

**Authors:** Nicole Scherr, Raphael Bieri, Sangeeta S. Thomas, Aurélie Chauffour, Nitin Pal Kalia, Paul Schneide, Marie-Thérèse Ruf, Araceli Lamelas, Malathy S. S. Manimekalai, Gerhard Grüber, Norihisa Ishii, Koichi Suzuki, Marcel Tanner, Garrett C. Moraski, Marvin J. Miller, Matthias Witschel, Vincent Jarlier, Gerd Pluschke, Kevin Pethe

**Affiliations:** 1 Swiss Tropical and Public Health Institute, Basel, 4051 Switzerland; 2 University of Basel, Basel, 4001 Switzerland; 3 Lee Kong Chian School of Medicine, Nanyang Technological University, Experimental Medicine Building, Singapore, 636921 Singapore; 4 CR7, INSERM, U1135, Centre d’Immunologie et des Maladies Infectieuses, CIMI, Team E13 (Bactériologie), Sorbonne Universités, UPMC Université Paris 06, Paris, 75005 France; 5 BASF SE, Ludwigshafen, 67063 Germany; 6 Red de Estudios Moleculares, AvanzadosInstituto de Ecología A. C., Xalapa, 91000 Veracruz Mexico; 7 School of Biological Sciences, Nanyang Technological University, Singapore, 637551 Singapore; 8 Department of Mycobacteriology, Leprosy Research Center, National Institute of Infectious Diseases, Tokyo, 189-0002 Japan; 9 Department of Clinical Laboratory Science, Faculty of Medical Technology, Teikyo University, Tokyo, 173-8605 Japan; 10 Department of Chemistry and Biochemistry, Montana State University, Bozeman, MT 59715 USA; 11 Department of Chemistry and Biochemistry, University of Notre Dame, Notre Dame, IN 46556 USA; 12 CNR-MyRMA, Bactériologie Hygiène, Hôpitaux Universitaires Pitie Salpêtrière-Charles Foix, Paris, 75013 France

## Abstract

*Mycobacterium ulcerans* is the causative agent of Buruli ulcer, a neglected tropical skin disease that is most commonly found in children from West and Central Africa. Despite the severity of the infection, therapeutic options are limited to antibiotics with severe side effects. Here, we show that *M. ulcerans* is susceptible to the anti-tubercular drug Q203 and related compounds targeting the respiratory cytochrome *bc*_*1*_*:aa*_*3*_. While the cytochrome *bc*_*1*_*:aa*_*3*_ is the primary terminal oxidase in *Mycobacterium tuberculosis*, the presence of an alternate bd-type terminal oxidase limits the bactericidal and sterilizing potency of Q203 against this bacterium. *M. ulcerans* strains found in Buruli ulcer patients from Africa and Australia lost all alternate terminal electron acceptors and rely exclusively on the cytochrome *bc*_*1*_*:aa*_*3*_ to respire. As a result, Q203 is bactericidal at low dose against *M. ulcerans* replicating in vitro and in mice, making the drug a promising candidate for Buruli ulcer treatment.

## Abstract (web summary)

*Mycobacterium ulcerans* is the causative agent of Buruli ulcer (BU). Existing anti-tubercular drugs have been used to treat the condition with varying success. Here, the authors show that a clinical-stage drug candidate for tuberculosis, Q203, is effective at killing *M. ulcerans* and is a promising therapeutic candidate for BU.

## Introduction

M*ycobacterium ulcerans* is the causative agent of Buruli ulcer (BU), a chronic necrotizing infection of the skin and the underlying tissue^1^. BU has been reported in more than 30 countries worldwide, but affects predominantly populations in West and Central Africa^2^, and is emerging in Australia^3^. Genomic analyses have shown that *M. ulcerans* has a common ancestor with *Mycobacterium marinum*^4^, a fish pathogen that occasionally causes granulomatous skin lesions in humans^5^. The ancestor of *M. ulcerans* acquired a virulence plasmid that carries the genes encoding the enzymatic machinery required for the synthesis of the cytotoxic macrolide toxin mycolactone^6^, which plays a key role in the chronic necrotizing pathogenesis of BU^7–9^. *Mycobacterium ulcerans* has subsequently undergone reductive evolution to become a niche-adapted organism with a 0.8 Mb smaller genome than *M. marinum*, and harbouring several hundred pseudogenes^10^. Two major lineages have been distinguished among *M. ulcerans* strains isolated from human BU lesions^11^. While the ancestral lineage found in patients from Asia, South America and Mexico is associated only sporadically with disease in humans, local incidence rates for BU caused by classical lineage strains in Africa and Australia can be as high as 1/200 per year^12^.

Until recently, antibiotic therapy of BU was thought to be ineffective, and wide surgical excision of BU lesions was the treatment of choice. Based on results with an experimental mouse infection model for BU, an 8 weeks daily treatment with rifampicin and streptomycin was clinically tested and implemented as standard of care^13,14^. Despite clinical efficacy^15^, nephrotoxic, hepatotoxic and ototoxic side effects^16,17^ combined with the requirement for daily injections advocate for alternative treatments. Repurposing of new tuberculosis drug candidates is an attractive approach to search for new treatment options for BU. However, most tuberculosis-active scaffolds have no, or limited activity against *M. ulcerans*^18^. Here, we show that reductive evolution drove the exquisite susceptibility of *M. ulcerans* to the imidazopyridine carboxamide (IPA) compound Q203^19^, a clinical-stage drug candidate for tuberculosis that targets the respiratory cytochrome *bc*_*1*_*:aa*_*3*_ (cyt-*bc*_*1*_*:aa*_*3*_). Recently, we demonstrated that the presence of the cytochrome-*bd* oxidase (cyt-*bd*), an alternate *bd*-type terminal oxidase, limits the sterilizing potency of Q203 in *Mycobacterium tuberculosis*^20^. *M. ulcerans* strains belonging to the classical lineage from African and Australian origin lost all terminal electron acceptors except the cyt-*bc*_*1*_*:aa*_*3*_, revealing the vulnerability of this target to chemical inhibition that can be exploited to develop potent drugs for BU.

## Results

### Cyt-*bd* is inactive in classical *M. ulcerans*

**Fig. 1:**
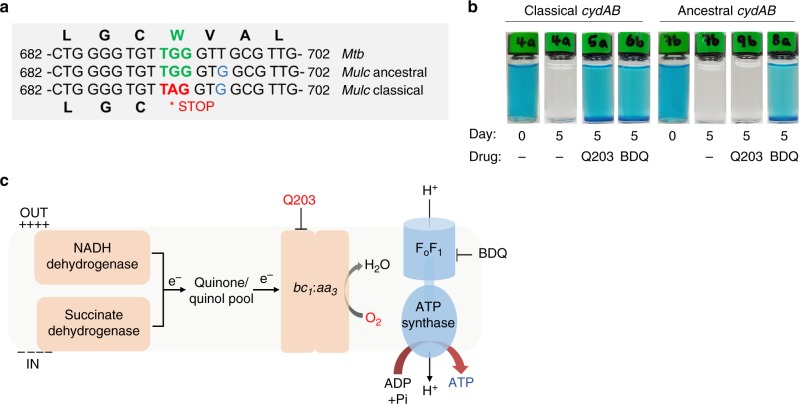
The cytochrome *bc*_*1*_*:aa*_*3*_ is the sole respiratory terminal oxidase in classical *M. ulcerans* strains. **a** A single-nucleotide polymorphism (692G>A) converting the tryptophan-encoding codon TGG into the stop codon TAG is present in *cydA* of all classical *M. ulceran*s strains. **b** The *cydAB* operon present in classical *M. ulcerans* strains does not encode a functional cyt-*bd*. Q203-treated *M. bovis* BCGΔ*cydAB* strains complemented with the *cydAB* operon from classical lineage, or from Japanese ancestral lineage strains were incubated with Q203 (100 nM) in sealed tubes containing 0.001% methylene blue used as an oxygen sensor. Pictures were taken immediately after closing the tubes (day 0), and 5 days after incubation at 37 °C. Bedaquiline (1 mM) and DMSO (−) were used as controls. **c** Oxidative phosphorylation pathway in classical *M. ulcerans* strains, the molecular targets of Q203 and bedaquiline (BDQ) are shown

Comparison of the sequences of the cyt-*bd*-encoding genes *cydAB* of *M. tuberculosis*, *M. marinum* and *M. ulcerans* revealed the presence of a nonsense mutation in the *cydA* sequences of the several 100 available genomes of African and Australian *M. ulcerans* strains^3,21–23^. A base substitution (692G>A) converted the tryptophan-encoding codon TGG into a stop codon (W231X) (Fig. 1a). In contrast to these classical lineage strains, the genomes of *M. marinum* and of ancestral Japanese *M. ulcerans* strains^24,25^ harbour a *cydA* gene encoding a full-length open-reading frame (Fig. 1a).

Functional assays were performed to verify the impact of the *cydA* W231X mutation on the activity of the cyt-*bd*. We made use of a *M. bovis* Bacillus Calmette–Guérin (BCG) strain deficient in the expression of cyt-*bd* (BCGΔ*cydAB*), in which oxygen consumption can be completely inhibited with drugs targeting the *cyt-bc*_*1*_*:aa*_*3*_^20^. The BCGΔ*cydAB* strain was transformed with the *cydAB* genes of either the classical or ancestral *M. ulcerans* lineages. Upon treatment with the cyt-*bc*_*1*_*:aa*_*3*_ inhibitor Q203, oxygen respiration was completely inhibited in the BCGΔ*cydAB* strain (Supplementary Fig. 1), as shown before^20^. Oxygen respiration was restored in the Q203-treated BCGΔ*cydAB* strain carrying the ancestral *cydAB* genes (Fig. 1b). Conversely, complementation with the *cydAB* genes of classical strain origin, having the *cydA* W231X mutation, failed to restore oxygen respiration (Fig. 1b). As a control, the drug bedaquiline (Sirturo®) that targets the mycobacterial F_1_F_o_ ATP synthase inhibited oxygen consumption in both strains (Fig. 1b). These results demonstrated that the cydA W231X polymorphism in the classical *M. ulcerans* strains has led to the loss of function of the cyt-*bd*. Furthermore, the genes encoding the anaerobic electron terminal acceptor nitrate reductase (Supplementary Fig. 2) and for fumarate reductase are missing in the genome of *M. ulcerans*, indicating that the cyt-*bc*_*1*_*:aa*_*3*_ is the only functional terminal electron acceptor in classical lineage *M. ulcerans* strains (Fig. 1c). This is in contrast to the ancestral *M. ulcerans* lineage and to *M. tuberculosis* that possess two and four terminal electron acceptors, respectively (Supplementary Fig. 3).

### *Mycobacterium ulcerans* is sensitive to drugs targeting the cyt-*bc*_*1*_*:aa*_*3*_

**Fig. 2:**
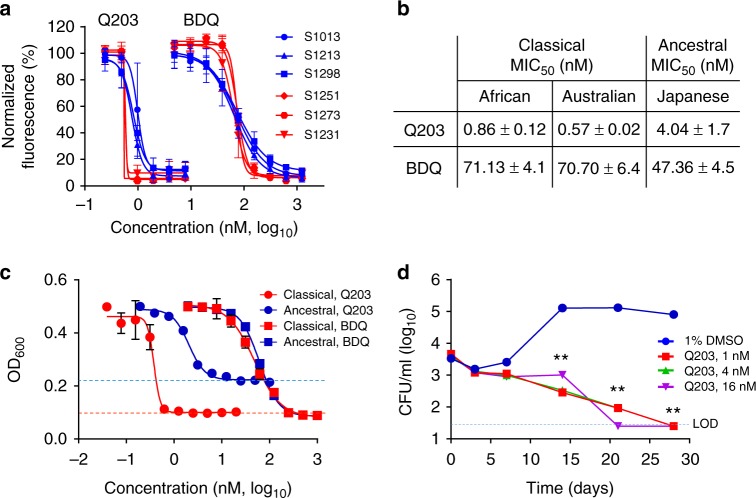
Potency of drugs targeting the cyt-*bc*_*1*_*:aa*_*3*_ against *M. ulcerans*. **a** Growth inhibitory activity of Q203 and bedaquiline (BDQ) against classical *M. ulcerans* clinical isolates from Africa (blue symbols) and Australia (red symbols) replicating in culture broth medium using a resazurin-based assay. Data are expressed as the mean ± s.d. of triplicates for each concentration. **b** Growth inhibitory activity (MIC_50_) of Q203 and BDQ against classical strains from African and Australian origin, and against ancestral strains from Japanese origin. MIC_50_ values are the average of three strains, each tested in duplicate. **c** Growth inhibitory activity of Q203 and bedaquiline (BDQ) against the classical strain S1013 (red symbols) and the ancestral strain S1325 (blue symbols) replicating in culture broth medium using a turbidity-based assay. Data are expressed as the mean ± s.d. of triplicates for each concentration. The red dotted line shows the bottom plateau for the dose–response curve for bedaquiline and Q203 against the classical strain; the blue dotted line shows the bottom plateau of the dose–response curve for Q203 against the ancestral strain. **d** Bactericidal potency of Q203 against the classical strain S1013 replicating in culture broth medium. Q203 was tested at a concentration of 1, 4, and 16 nM in triplicate and repeated once. The dotted line represents the limit of detection. **P value <0.001, using the Student's *t* test, between the untreated control group (1% DMSO) and Q203 at either 1, 4 or 16 nM

The *M. ulcerans* cyt-*bc*_*1*_ complex subunit QcrB has 90.3% sequence identity with the *M. tuberculosis* counterpart, and possesses an additional 10 residues, _206_GGVGDDCTAA_215_, located in an outside loop (Supplementary Fig. 4). Furthermore, the residues for which substitutions are associated with resistance to IPA compounds in *M. tuberculosis*^19,26^ are all conserved (Supplementary Fig. 4). The comparison of the 3-dimensional model of QcrB between *M. tuberculosis* and *M. ulcerans* revealed an overall similar topology (Supplementary Fig. 5). The model revealed that the additional 10-residue region in the *M. ulcerans* QcrB is unlikely to influence the interaction between the target and the IPA compounds. This is because the distance between the region _206_GGVGDDCTAA_215_ to all residues known to confer resistance to IPA derivatives in *M. tuberculosis* is more than 25 Å, a distance greater than the 13 Å length of Q203, one of the largest IPA derivatives. These observations suggested that the lead optimization of the IPA series that resulted in the anti-tuberculosis drug candidate Q203 may also be valid for *M. ulcerans*. Profiling of 87 IPA compounds for activity against the classical *M. ulcerans* strain S1013 using a resazurin-based minimum inhibitory concentration (MIC) assay (Table 1 and Supplementary Table 1) revealed a comparable structure–activity relationship to the trend observed in *M. tuberculosis*^27^. Small residues such as halogen, methyl or ethyl were tolerated on the imidazopyridine core, while the optimum activity of the substitution pattern depended on the amide residue (Supplementary Table 1). Q203 was the most active derivative with an outstanding minimum inhibitory concentration (MIC_50_) below 1 nM in all six tested *M. ulcerans* classical lineage isolates of African and Australian origin (Fig. 2a, b). Of note, two additional advanced preclinical IPA drug candidates, ND-10885^28^ and ND-11176^29^, were also potent against *M. ulcerans* (Table 1). The MIC_50_ values of Q203 against Japanese ancestral lineage isolates encoding a functional cyt-*bd* were 4- to 8-fold higher compared to the classical strains (Fig. 2b). In contrast, classical and ancestral lineage isolates were equally sensitive to bedaquiline (Fig. 2b). The sub-nanomolar potency of Q203 against the classical lineage and the reduced potency against the ancestral lineage was reconfirmed using two assays determining directly the increase in bacterial biomass for the prototype isolates S1013 (classical) and S1325 (ancestral) (Fig. 2c, supplementary Fig. 6). Interestingly, Q203 inhibited the growth of the ancestral strain only partially, as witnessed by the high level of the bottom plateau in the optical density-based assay (Fig. 2c), and the limited effect on the size of the bacterial pellets in the pellet-formation assay (Supplementary Fig. 6).

**Table 1:** Activity of IPA derivatives against the classical *M. ulcerans* strain S1013

The potency of the IPA derivatives was tested in dose–response against *M. ulcerans* S1013 replicating in culture broth medium. Compounds **5**, **13**, **31** and **80** are representatives of different activity groups listed in detail in Supplementary Table 1

*cLogP calculated with PerkinElmer ChemDraw Professional 16.0.1.4

**Fig. 3:**
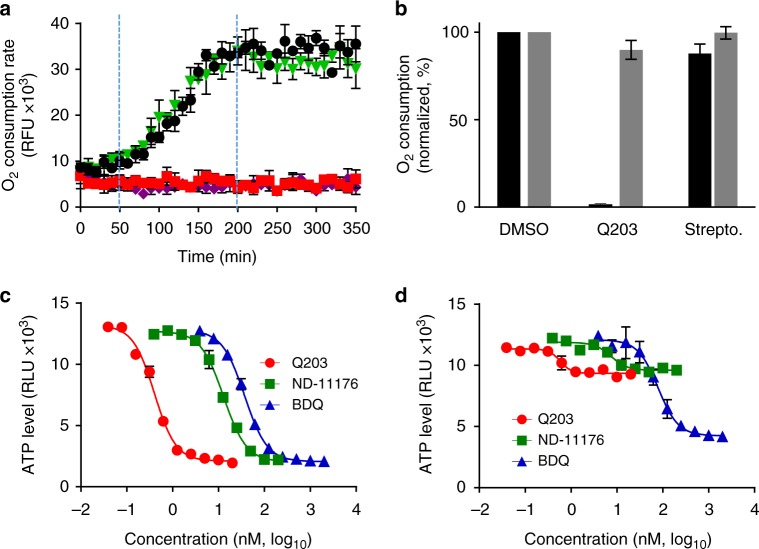
Inhibition of cyt-*bc*_*1*_*:aa*_*3*_ arrests oxidative phosphorylation in classical *M. ulcerans*. **a** Oxygen consumption rates in the classical lineage *M. ulcerans* strain S1013 treated with Q203 (5 nM, red squares), ND-11176 (50 nM, purple diamonds), streptomycin (5000 nM, green triangles) or DMSO (1%, black circles) was measured using the MitoXpress® Xtra-oxygen probe. Fluorescence (Ex_380 nm_, Em_650 nm_) was measured over 350 min. The experiment was performed in triplicate and repeated two times. **b** Effect of Q203 (5 nM) or streptomycin (5000 nM) treatment on the oxygen consumption rate in the classical strain S1013 (dark grey bars) and the ancestral strain S1325. **c**, **d** Quantification of intracellular ATP levels in the classical strain S1013 (**c**) or in the ancestral strain S1325 (**d**) treated with a dose range of Q203, ND-11176 or bedaquiline (BDQ) for 24 h. Every concentration was tested in triplicate and the assay repeated once

In contrast to *M. tuberculosis*, in which Q203 is only bacteriostatic^20^, Q203 was bactericidal against the classical strain S1013 replicating in vitro (Fig. 2d), confirming that the absence of an alternate *bd*-type terminal oxidase in the classical *M. ulcerans* lineage sensitizes the bacterium to killing by Q203. To demonstrate that IPA compounds kill *M. ulcerans* by inhibiting oxidative phosphorylation, the effect of Q203 on oxygen respiration was measured. Q203 treatment inhibited the oxygen consumption rate (OCR) by 98.5% (Fig. 3a, b), whereas similar treatment had limited effect against the ancestral strain S1325 that possesses a functional cyt-*bd* (Fig. 3b). The near-complete inhibition of oxygen respiration in the classical strain S1013 correlated with a rapid and profound ATP depletion at a half-maximal inhibitory concentration (IC_50_) of 670 pM, a concentration 100-fold lower compared to bedaquiline (Fig. 3c). ATP depletion was rapid and sustained over time (Supplementary Fig. 7). Conversely, Q203 had a limited effect on ATP levels in the ancestral strain S1325 (Fig. 3d). The IPA derivative ND-11176 inhibited the OCR (Fig. 3a) and ATP synthesis (Fig. 3c) in the classical lineage strain S1013 to the same extent as observed with Q203, showing that oxidative phosphorylation inhibition is not restricted to Q203 but extends to other preclinical IPA drug candidates. Together, these findings demonstrate that the absence of a functional cyt-*bd* in classical *M. ulcerans* strains sensitized the bacteria to drugs targeting the cyt-*bc*_*1*_*:aa*_*3*_ terminal oxidase. Relying solely on the cyt-*bc*_*1*_*:aa*_*3*_ terminal oxidase to perform oxidative phosphorylation, these strains are unable to reroute the electron flow to an alternate terminal electron acceptor, and to mitigate the effect of Q203 as observed in *M. tuberculosis*^20^.

### Q203 has a low propensity to select for resistance in vitro

Propensity to elicit the development of spontaneous resistance is a critical factor for the development of a novel antibacterial. Mutant selection experiments on agar plates containing 10 nM of Q203 determined the spontaneous rate of mutation in the range of 5.6 × 10^−9^, indicating a very low probability of emergence of resistant mutants. All the analysed resistant mutants (14/14) had a non-synonymous single-nucleotide polymorphism in *QcrB* leading to the amino acids substitutions T313A, M352V, M352T, or W322C (Supplementary Table 2). Substitution of these amino acids has been reported to confer resistance to Q203 or other IPA derivatives in *M. tuberculosis*^19,26^. The escape mutant harbouring the mutation M352T in QcrB was lost shortly after selection due to its inability to grow in liquid broth medium. Sequencing of *cydA* in the 13 other spontaneous-resistant mutants revealed that the base substitution G692A was still present, suggesting that the reversion to a functional cyt-*bd* under Q203 pressure has a low probability to be selected (<4.0 × 10^−10^).

### Q203 eradicates *M. ulcerans* infection in a mouse model

**Fig. 4:**
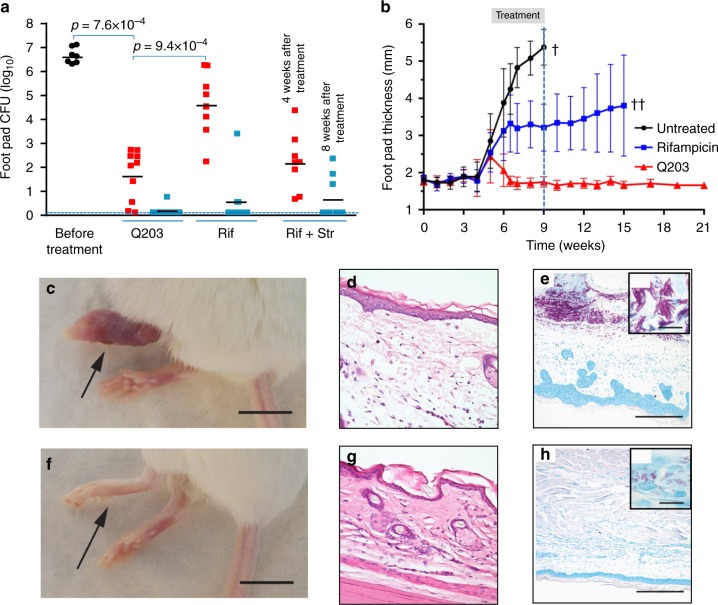
Efficacy of Q203 in the mouse footpad infection model of Buruli ulcer. **a** Bacterial loads were enumerated in the footpads of mice infected with *M. ulcerans* Cu001 after 4 and 8 weeks of daily treatment. Q203, rifampicin (Rif) and streptomycin (Str) were used at 0.5, 10 and 150 mg/kg body weight, respectively. Q203 and rifampicin were administered orally, streptomycin was given by the sub-cutaneous route. Ten mice per group and per time point were used. Data are expressed as mean ± s.d. Statistical analysis was performed using two-sided Mann–Whitney *U* test. **b** Footpad thickness was followed over time after infection with *M. ulcerans* S1013 on day 0. Q203 and rifampicin were administered orally three times per week at 0.5 and 10 mg/kg body weight, respectively, for 4 weeks (starting from week 5; treatment interval is boxed). Mean values with standard deviation are shown. The single cross symbol represents all 12 animals from the untreated group had to be euthanized at week 9 post infection due to severe disease progression. The double cross symbol represents all remaining eight animals from the rifampicin-treated group had to be euthanized at week 15 post infection due to unfavourable progression in the majority of the animals (5/8). **c**, **f** Appearance of infected foot at week 9 treated with the vehicle control (**c**) or with Q203 (**f**). Black arrows shows the site of infection. **d**–**g** Oedema formation and tissue necrosis in footpads from untreated control animals (**d**) and complete lack of oedema and tissue necrosis in Q203-treated animals (**g**) after completion of treatment at week 9. **e**–**h** Large extracellular clusters of solid-stained AFB embedded in the necrotic lesions in footpads of the untreated mice (**e**) and small numbers of AFB with beaded, not solid-stained appearance in the Q203-treated mice (**h**) at week 15. Scale bars: **c**, **f**: 1 cm; **d**, **g**: 80 μm; **e**, **h**: 200 μm; inlets in **e**, **h**: 10 μm

The high activity of Q203 against classical *M. ulcerans* strains in vitro translated into high treatment efficacy in the well-established mouse footpad infection model of BU. In a first set of experiments, the potency of Q203 was compared to that of rifampicin or rifampicin + streptomycin. Given the favourable pharmacokinetic properties of Q203^19^ that were confirmed in this study (Supplementary Table 3), we estimated that Q203 at 0.5 mg/kg body weight would be an appropriate dose to achieve high efficacy in vivo. Daily drug treatment was initiated 5 weeks after infection with the classical African strain Cu001^30^, a time point at which animals had developed a swelling of their inoculated footpad. After 4 weeks of daily treatment (5 times per week; orally for Q203 and rifampicin and sub-cutaneously for streptomycin), the bacterial load in the animals treated with Q203 0.5 mg/kg body weight was reduced by more than 99.99% compared to the untreated control group (Fig. 4a). Furthermore, the bacterial load in the food pad of Q203-treated animals was 1000-fold lower compared to the group treated with rifampicin 10 mg/kg body weight (Fig. 4a), showing that Q203 had a superior activity than rifampicin when given at a dose 20-fold lower. Furthermore, Q203 at 0.5 mg/kg body weight was as good as the clinically used drug combination rifampicin 10 mg/kg body weight + streptomycin 150 mg/kg body weight in reducing bacterial load at week 4 post treatment (Fig. 4a). After 8 weeks of daily treatment, Q203 had eradicated *M. ulcerans* infection in 90% (9/10) of the mice, whereas only five colonies grew from the footpad lysate of the last animal (Fig. 4a). This level of efficacy at 8 weeks compared favourably with the rifampicin and rifampicin + streptomycin treatment that eradicated infection in 75% (6/8) and 66.6% (6/9) of the treated mice, respectively (Fig. 4a). Given the stability and long half-life of Q203^19^, we next tested whether intermittent administration of Q203 would resolve a BU infection in mice. Five weeks after infection with the classical strain S1013, when the mice started to show swelling of the infected feet (Fig. 4b), treatment was initiated by oral administration of Q203 at 0.5 mg/kg body weight three times per week for 4 weeks. During that time, a strong increase in footpad thickness was observed for the untreated control animals (Fig. 4b, c). Histopathological analyses of footpads from these control animals revealed oedema formation and massive tissue necrosis (Fig. 4d). Due to severe progression of the pathogenesis, the control animals had to be euthanized at week 9 post infection. In contrast, a complete regression of the footpad swelling was observed in the Q203-treated animals as early as 10 days after treatment initiation (Fig. 4b). Footpad thickness in the Q203-treated mice returned to normal levels (Fig. 4f) and did not increase further for the entire observation period of 6 months post treatment (Fig. 4b). Strikingly, the footpads of the Q203-treated mice were completely devoid of oedema and tissue necrosis after completion of treatment (Fig. 4g). While large extracellular clusters of solid-stained acid-fast bacilli (AFB) embedded in the necrotic lesions were observed in footpads of the untreated control mice (Fig. 4e), only small numbers of AFB with beaded, not solid-stained appearance were found in the Q203-treated mice at week 15 (Fig. 4h), indicating loss of bacterial viability^31^. The absence of viable bacteria in Q203-treated animals was confirmed 5 weeks after completion of treatment (Supplementary Fig. 8). Conversely, intermittent administration of rifampicin at 10 mg/kg body was unable to stop disease progression completely, as witnessed by continuing footpad swelling (Fig. 4b) and reflected by the presence of large numbers of colony-forming units (CFU) 5 weeks after completion of treatment in 5/8 mice (Supplementary Fig. 8). This result illustrates the potential advantage of Q203 over drugs with a shorter half-life to simplify BU treatment. It is interesting to note that, whereas a dose of 0.5 mg/kg body weight of Q203 given three times per week was sufficient to eradicate a BU infection in the mouse model, a dose of 2 mg/kg body weight administered following a comparable regimen had no effect in an experimental mouse model of tuberculosis^20^, confirming the exceptional susceptibility of the classical lineage of *M. ulcerans* to the IPA compounds.

## Discussion

While traditionally surgical excision has been the only treatment recommended for BU, the World Health Organization published in 2004 a provisional guidance recommending a combination therapy with daily administration of rifampicin and streptomycin for a period of 8 weeks^2^. Despite clinical efficacy, administration of streptomycin through injections has a negative impact on patient acceptance and adherence and is frequently associated with permanent ototoxicity and nephrotoxicity^16,17^. Therefore, replacement of streptomycin with oral clarithromycin is currently considered by the World Health Organization (WHO) Technical Advisory Group on BU. Rifampicin is currently the most effective drug against *M. ulcerans* and essential for successful treatment. In case of emergence of rifampicin resistance in *M. ulcerans*, no antibiotics are currently available as substitutes. Our study indicates that drugs targeting the respiratory cyt-*bc*_*1*_*:aa*_*3*_ are promising agents to treat BU. Strains of the classical *M. ulcerans* lineage are exquisitely sensitive to Q203 because their respiration relies exclusively on the cyt-*bc*_*1*_*:aa*_*3*_ terminal oxidase. Strains belonging to the classical lineage are responsible for the BU foci in Africa and Australia that are typically characterized by high local prevalence^12^. In contrast, the ancestral lineage is responsible for only scattered, sporadic BU cases recorded in Asia and the Americas^12^. Of the 58,894 BU cases reported between 2002 and 2016, it is estimated that the vast majority (>97%) is caused by classical lineage strains^12^ lacking a functional cyt-*bd*.

Ongoing reductive evolution has been associated with resistance to anti-tubercular drugs due to possible loss of targets^18^ or enzymes required for prodrug bio-activation^32,33^. This study demonstrates that reductive evolution can drive hyper-susceptibility to certain classes of drugs by eliminating metabolic redundancy. In the particular case of the oxidative phosphorylation pathway, the loss of alternate terminal electron acceptors makes the cyt-*bc*_*1*_*:aa*_*3*_ absolutely essential for survival, and therefore an excellent drug target. The clinical-stage drug Q203 has a strong bactericidal activity against classical *M. ulcerans* strains both in vitro and in vivo, and could both simplify and shorten BU treatment. In the mouse infection model, oral administration of Q203 three times per week for 4 weeks was highly effective. Given the high potency and long half-life of Q203, an even simpler treatment regimen may be possible. It is interesting to note that since Q203 and bedaquiline have a comparable long half-life, they could be combined to develop a radically simplified treatment regimen for BU.

Together, our data demonstrate that drugs targeting the cyt-*bc*_*1*_*:aa*_*3*_ complex, such as the IPAs, and exemplified by the clinical candidate Q203, are promising drug candidates for BU. The natural loss of cyt-*bd* function argues that Q203 may have a higher efficacy and safety margin for BU treatment in Africa and Australia than for tuberculosis treatment; assumptions that should be tested in human clinical trials. The concept may also apply to *Mycobacterium leprae*, another mycobacterial species that does not possess as well the cyt-*bd*-encoding genes^34^.

## Methods

### Bacterial strains and growth conditions

*M. ulcerans* strains were isolated from BU lesions of patients from Cameroon (strains S1013, S1213 and S1298), Cote d’Ivoire (Cu001), Australia (strains S1251, S1273 and S1231) or Japan (strains S1324, S1325 and S1326). Bacteria were cultivated at 32 °C in BacT/Alert culture bottles supplemented with enrichment medium (bioMérieux). For in vitro compound testing, bacteria were grown either in Middlebrook 7H9 medium (Difco) or in Middlebrook 7H10 agar (Difco), supplemented with 10% (vol/vol) Middlebrook OADC supplement.

### MIC_50_ determination

MIC_50_ was determined using a resazurin microtiter assay^35^. Cultures were incubated at an initial optical density 600 nm (OD_600_) of 0.02 with a dose range of antibiotics (2-fold dilution range) at 32 °C for 8 days. End-point fluorescence reading (Ex_540 nm_/Em_588 nm_) was done after incubation with 10% (vol/vol) resazurin solution (0.125 mg/mL, Sigma) for 24 h. Alternatively, MIC_50_ was determined by the broth microdilution method^36^ adapted for *M. ulcerans*. A volume of 150 μl of *M. ulcerans* culture (final OD_600_ of 0.02) was added to each well containing a dose range of antibiotics, and the assay plates were incubated at 32 °C for 15 days. OD_600_ values were recorded using a Cytation 5 Cell Imaging Multi-Mode Reader, and MIC_50_ curves were plotted using the GraphPad Prism 5 software.

### MBC determination

*M. ulcerans* S1013 at an OD_600_ of 0.01 was incubated with and without Q203 at 32 °C. Q203 potency was tested at a concentration of 1, 4 and 16 nM. Viability was determined by CFU determination on agar plates over a period of 90 days.

### Expression of the *M. ulcerans cydAB* operons in *M. bovis* BCG

Two complementation plasmids were created by incorporating the *cydAB* genes from the African or Japanese *M. ulcerans* strains into the pmv306 vector. The plasmids were electroporated in the *M. bovis* BCGΔ*cydAB*^20^. Transformants were selected on 7H10-OADC agar plates containing 20 µg/mL of kanamycin.

### Oxygen consumption assays

The methylene blue assays and the MitoXpress oxygen consumption assay were used to measure oxygen consumption in mycobacteria as described before^20^. Briefly, mycobacteria were grown to an OD_600_ of 0.3 in Middlebrook 7H9 media. The cultures were washed twice and resuspended in Middlebrook 7H9 medium without glycerol and incubated with Q203 (100 nM), bedaquiline (1 µM), or 1% dimethyl sulfoxide (DMSO) (solvent control). The cultures were then transferred to 2 mL glass tubes containing methylene blue at a final concentration of 0.001%. The tubes were closed tightly and incubated at 37 °C for 5 days under anaerobic conditions to avoid oxygen leakage within the tubes. Pictures were taken immediately after incubation, and 5 days post incubation.

For the MitoXpress oxygen consumption assay, *M. ulcerans* strain S1013 was grown to an OD_600_ of 0.9 in Middlebrook 7H9-ADS medium. Cultures were pre-incubated with Q203 (5 nM), streptomycin (5 µM) or 1% DMSO (solvent control) for 6 h. One hundred and fifty microliters of each culture were transferred to black, clear-bottom 96-well plates containing 10 µL of the MitoXpress probe (MitoXpress Xtra-Oxygen consumption assay, Luxcel Biosciences). Each well was overlaid with high-sensitivity oil to prevent oxygen diffusion. Time resolved fluorescence reading (Ex_380 nm_/Em_650 nm_) was recorded on a BioTek Cytation 5 reader for 5 h. Lifetime calculation of each condition was computed, and the OCR was determined from the slope of the lifetime graphs.

### Intracellular ATP quantification

ATP levels were measured using the BacTiter-Glo™ Microbial Cell Viability Assay (Promega). ATP levels were quantified at 24 and 72 h post treatment by adding an equal volume of Bactiter-Glo reagent. Luminescence reading was recorded on a BioTek CYTATION 3 reader.

### Mouse footpad infection model

Efficacy testing by CFU determination. The study was granted ethical approval by the animal welfare committee of the Sorbonne Universities, Faculté de Médecine Pierre et Marie-Curie (Authorization Number: APAFIS 9576-2017030117176185 v2). Four- to six-week-old BALB/c mice (Janvier Labs, Le Genest Saint-Isle, France) were inoculated in the left hind footpad with 30 μL of a bacterial suspension containing 2 × 10^4^ CFU of *M. ulcerans* Cu001. Seven weeks after inoculation, swelling in the inoculated footpad confirmed that the infection was well established. Mice were randomly allocated to one of the either following groups: untreated control (receiving vehicle control only), Q203-treated (0.5 mg/kg body weight), rifampicin (10 mg/kg body weight) or rifampicin + streptomycin (10 mg/kg body weight and 150 mg/kg body weight, respectively). Q203 and rifampicin were administered daily (5 times per week for 8 weeks) by oral dosing, whereas streptomycin was administered sub-cutaneously. Ten animals per condition and per time point were sacrificed for CFU determination in their infected footpad, unless less animal were available due to unforeseen conditions. For the week 4 data point, only nine and eight animals could be processed for CFU determination in the rifampicin and rifampicin + streptomycin groups, respectively, because the other animals died during the gavaging period. For the same reason, only eight and nine animals could be processed for CFU determination in the rifampicin and rifampicin + streptomycin groups, respectively, at the week 8 data point. For CFU determination, the tissues from the infected footpad were removed aseptically and homogenized in 2 mL Hank’s balanced salt solution. For the treated groups, the entire volumes were spread on 10 Lowenstein–Jensen medium tubes (0.2 mL of suspension per tube). For the untreated controls, footpad homogenates were serial diluted and plated on Lowenstein–Jensen medium. The tubes were incubated at 30 °C for 90 days before CFU determination.

Efficacy testing of intermittent dosing by gross pathology and histopathology analysis. The study was carried out in accordance with the Rules and Regulations for the Protection of Animal Rights (Tierschutzgesetz SR455) of the Swiss Federal Food Safety and Veterinary Office. The protocol was granted ethical approval by the Veterinary Office of the county of Vaud, Switzerland (Authorization Number: 2657). Forty eight-week-old female BALB/c mice (Harlan) received an infection inoculum of 6 × 10^3^ classical *M. ulcerans* S1013 in a volume of 30 µL into the hind left footpad. Mice were randomly assigned to either the untreated, Q203-treated, or rifampicin-treated groups (12 animals per group). The course of infection was followed by measurement of the footpad thickness with a calliper. Oral treatment was started 5 weeks after infection. Four mice were euthanized at week 5 post infection to determine the bacterial load in the inoculated footpad before treatment initiation. Mice received for a period of 4 weeks on 3 days per week 0.5 mg/kg body Q203 suspended in 20% d-α-tocopherol polyethylene glycol 1000 succinate (Sigma, 57688) in water containing 1% DMSO (Sigma, D2650), rifampicin, or the vehicle control only. At week 4 post treatment (week 9 post infection), four animals for the Q203- and rifampicin-treated groups, and the 12 animals from the untreated group were euthanized for analysis. All the animals from the untreated group had to be euthanized at this time point due to the severe progression of the infection. For histopathological analysis, mouse feet were removed above the ankle, and fixed at room temperature during 48 h in 10% neutral-buffered formalin solution (Sigma). The feet were decalcified in formic acid bone decalcifier (Immunocal^TM^, StatLab) for 6 days at room temperature and subsequently transferred to 70% ethyl alcohol. After dehydration and paraffin embedding, 5 μm thin sections were cut, de-paraffinized, rehydrated, and stained according to WHO standard protocolswww.who.int/buruli/laboratory_diagnosis/en/ with haematoxylin/eosin (Sigma) or Ziehl–Neelsen/methylene blue (Sigma). At week 15 post infection (week 6 post treatment), four animals from the Q203-treated group and eight animals from the rifampicin-treated groups were euthanized for CFU determination. All remaining animals from the rifampicin-treated group had to be euthanized at this time point due to unfavourable disease progression.

### Selection of spontaneous Q203-resistant mutants

*M. ulcerans* S1013 was spread onto 7H11-OADC agar plates containing 10 nM of Q203 and incubated at 32 °C for 10 weeks. Incubation of the plates for an additional 6 weeks did not yield further colonies. Fourteen colonies, picked from different plates, were grown in 7H9-ADS-Glycerol-Tween 80 medium supplemented with 2 nM of Q203, and subjected to sequencing of *QcrB* and *cydA* genes. The resistance phenotype to Q203 was confirmed by testing susceptibility to a dose range of Q203.

## Supplementary information

Supplementary Information

Reporting Summary

## Data Availability

The data that support the findings of this study are available from the corresponding authors upon reasonable request.
