# Supplementary material for: Targeting the Mycobacterium ulcerans cytochrome bc1:aa3 for the treatment of Buruli ulcer
Source: Nat Commun. 2018 Dec 18;9:5370. doi: 10.1038/s41467-018-07804-8 (PMC6299076; doi:10.1038/s41467-018-07804-8)
Supplement: Supplementary file 1 — Supplementary Information [file 41467_2018_7804_MOESM1_ESM.pdf]

SUPPLEMENTARY INFORMATION

Targeting the Mycobacterium ulcerans cytochrome bc_1:aa_3 for the treatment of Buruli ulcer.

Nicole Scherr *et al.*

Supplementary Figures 1-8

Supplementary Tables 1-3

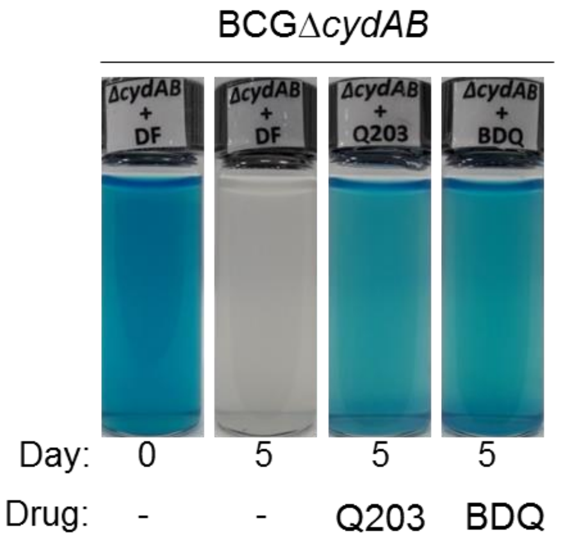

Supplementary Figure 1. Oxygen respiration is inhibited by Q203 in *M. bovis* BCG Δ*cydAB*. BCG Δ*cydAB* was incubated with Q203 (100 nM) in sealed tubes containing 0.001% methylene blue used as an oxygen sensor. Pictures were taken immediately after closing the tubes (day: 0), and 5 days (day: 5) after incubation at 37°C. Bedaquiline (BDQ, 1 mM) and DMSO (-) were used as controls.

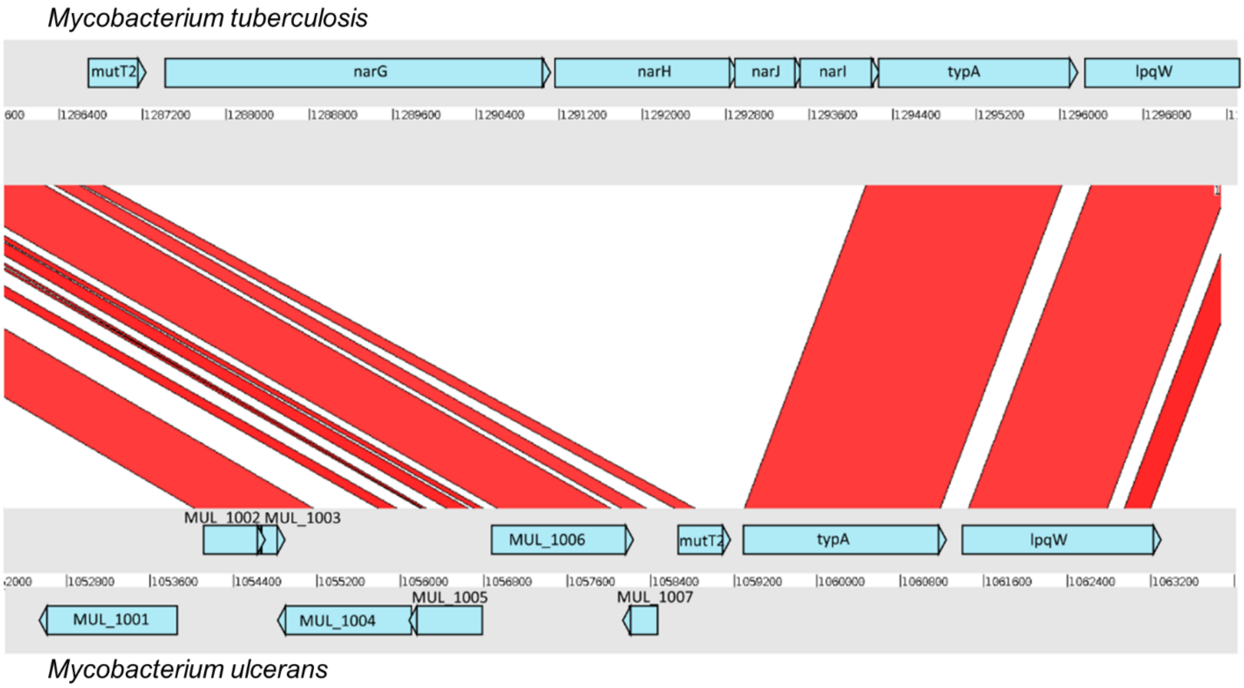

Supplementary Figure 2. The nitrate reductase-encoding operon *narGHJI* is missing in the genome of *M. ulcerans*. The genomic region of *Mycobacterium tuberculosis* strain H37Rv (NC_000962) comprising the narGHJ genes was compared to the same region of *Mycobacterium ulcerans* Agy99 (CP000325) using the big_blast.pl script. The comparisons were visualized with the Artemis Comparison Tool (ACT). Red bars are connecting matching regions. Absence of the *narGHJI* operon was confirmed in all other analyzed *M. ulcerans* genomes.

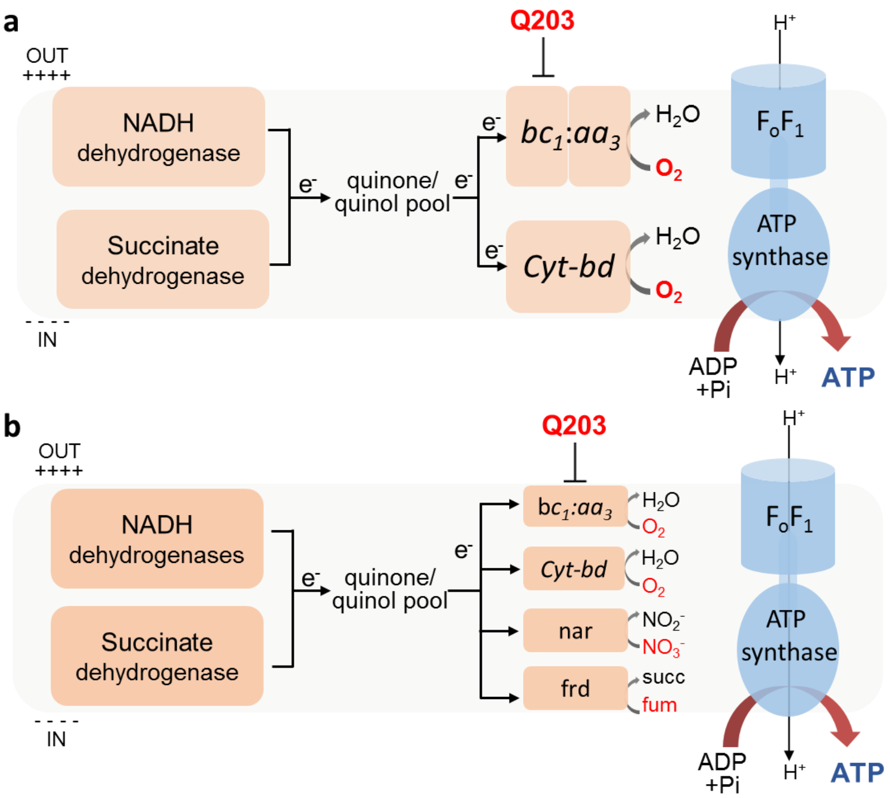

Supplementary Figure 3. Oxidative phosphorylation pathway in ancestral *M. ulcerans* (a) and in *M. tuberculosis* (b). nar: nitrate reductase, frd: fumarate reductase.

```
Mtb_QcrB    MSPKLSPPNIGEVLARQAEDIDTRYHPSAALRRQLNKVFPTHWSFLLGEIALYSFVVLLI 60
Mulc_QcrB   MSPKLSPPKIGDVLARQAEDIDTRYHPAAALRRQFNKVFPTHWSFLLGEVALYSFIVLLI 60
            ********:**:***************:******:**************:*****:****

Mtb_QcrB    TGVYLTLFFDPSMVDVTYNGVYQPLRGVEMSRAYQSALDISFEVRGGLFVRQIHHWAALM 120
Mulc_QcrB   TGVYLTLFFDPSMMDVTYNGVYQPLRGVEMSKAYASALDISFEVRGGLFVRQVHHWAALM 120
            *************:*****************:** *****************:*******

Mtb_QcrB    FAAAIMVHLARIFFTGAFRRPRETNWVIGSLLLILAMFEGYFGYSLPDDLLSGLGLRAAL 180
Mulc_QcrB   FAAAIMVHLARIFFTGAFRRPREANWIIGSLLLILAMFEGYFGYSLPDDLLSGIGLRAAL 180
            ***********************:**:**************************:******

Mtb_QcrB    SSITLGMPVIGTWLHWALFGGDFPG----------TILIPRLYALHILLLPGIILALIGL 230
Mulc_QcrB   SSITLGMPVIGTWLHWALFGGDFPQGGVGDDCTAAGYIIPRMYSLHILLLPGIILALIGM 240
            ************************             :***:*:***************:

Mtb_QcrB    HLALVWFQKHTQFPGPGRTEHNVVGVRVMPVFAFKSGAFFAAIVGVLGLMGGLLQINPIW 290
Mulc_QcrB   HMALVWFQKHTQFPGPGRTEHNVVGVRVMPVFAVKSGAFFAAITGVLGLMGGLLQINPIW 300
            *:*******************************.*********.****************

Mtb_QcrB    NLGPYKPSQVSAGSQPDFYMMWTEGLARIWPPWEFYFWHHTIPAPVWVAVIMGLVFVLLP 350
Mulc_QcrB   NLGPYKPAHVSAGSQPDFYMMWTEGLARIWPPWEFYFWHHTIPAPVWVALIMGLIFMLLI 360
            *******::****************************************:****:*:**

Mtb_QcrB    AYPFLEKRFTGDYAHHNLLQRPRDVPVRTAIGAMAIAFYMVLTLAAMNDIIALKFHISLN 410
Mulc_QcrB   VYPFLEKRFTGDYAHHNLLQRPRDAPVRTAVGAMAISFYMLLTLAAMNDIIALKFHISLN 420
            .***********************.*****:*****:***:*******************

Mtb_QcrB    ATTWIGRIGMVILPPFVYFITYRWCIGLQRSDRSVLEHGVETGIIKRLPHGAYIELHQPL 470
Mulc_QcrB   ATTWIGRIGMVILPPFVYFISYRWSIGLQRSDRAVLEHGIETGIIKRLPHGAYIELHQPL 480
            ********************:***.********:*****:********************

Mtb_QcrB    GPVDEHGHPIPLQYQGAPLPKRMNKLGSAGSPGSGSFLFADSAAEDAALREAGHAAEQRA 530
Mulc_QcrB   GPVDEHGHPLPLDYQGAPLPKRMNKLGSAGSPGSGSFLTADPASEDAALREAGHAAEHRA 540
            *********:**:************************* ** *:*************:**

Mtb_QcrB    LAALREHQDSIMGSPDGEH-- 549
Mulc_QcrB   LTALREYQDSLNETSNGEGDH 561
            *:****:***:  : :**
```

Supplementary Figure 4. Alignment of the *M. tuberculosis* and *M. ulcerans* QcrB. The sequences of QcrB from *M. tuberculosis* (Mtb_QcrB) and *M. ulcerans* (Mulc_QcrB) were aligned using the Clustal Omega program. The ten additional amino acids found in the *M. ulcerans* qcrB are boxed. The residues for which substitutions are reported to be associated with resistance to the IPA series are highlighted in red.

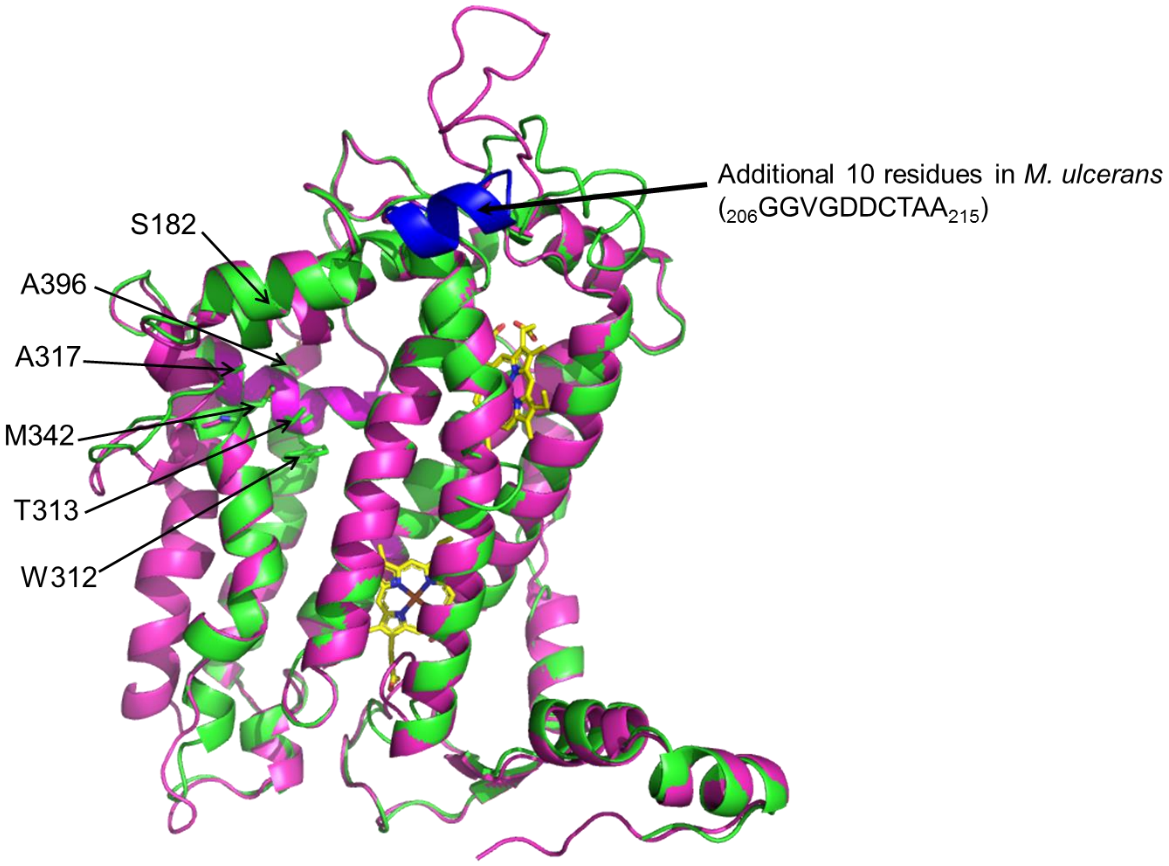

Supplementary Figure 5. Three-dimensional model comparison of QcrB between *M. tuberculosis* (*green*) and *M. ulcerans* (*magenta*). The SWISS-MODEL server was used to create the 3D models using homology modelling. For *M. ulcerans*, the *Rhodobacter sphaeroides* crystal structure (PDB ID: 2QIY) was used as a template. A model was generated with sequence coverage of 80% and GMQE (Global Model Quality Estimation) of around 0.47. For *M. tuberculosis*, the *Paracoccus denitrificans* crystal structure (PDB ID: 2YIU) was used as template. A model was generated with sequence coverage of 77% and GMQE of around 0.43. The heme groups for the *M. ulcerans* QcrB were manually modelled based on the template structure. The *M. ulcerans* model overlaps nicely with the *M. tuberculosis* model with a root mean square deviation of 0.32 Å. The additional 10 residues present in the *M. ulcerans* sequence (*blue*), and the amino acids for which substitution are associated with resistance to the IPA series in *M. tuberculosis*, are highlighted.

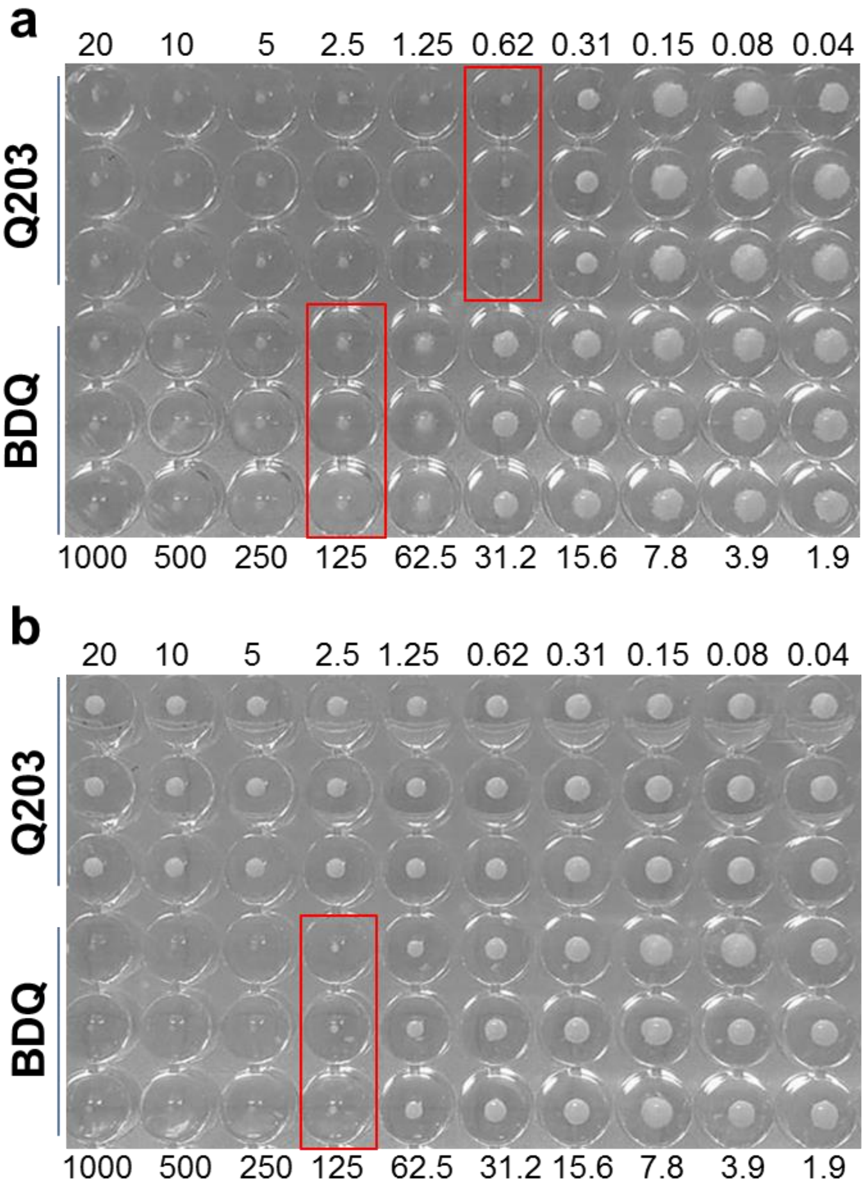

Supplementary Figure 6. MIC of Q203 and bedaquiline in *M. ulcerans* determined by a visual pellet-formation assay. The classical *M. ulcerans* strain S1013 (a) and the ancestral *M. ulcerans* strain S1325 (b) were treated with 2-fold serial dilutions of Q203 or bedaquiline (BDQ) for 15 days before pictures of the plates were taken. Q203 was tested from 20 to 0.04 nM and BDQ was tested from 1000 to 1.9 nM. Each drug concentration was tested in triplicate.

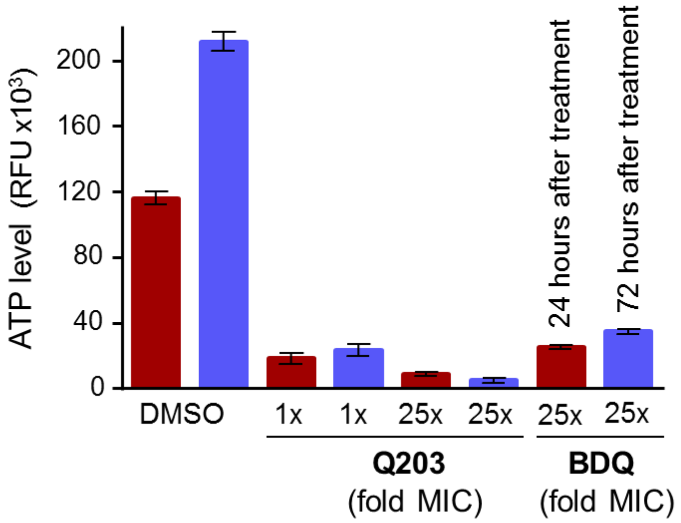

Supplementary Figure 7. Q203 triggers a profound and sustained ATP depletion in classical *M. ulcerans* strains. *M. ulcerans* S1013 was treated with Q203 at 1x or 25x its MIC for 24 hours (red bars) and 72 hours (blue bars) before quantifying intracellular ATP using the BacTiter-Glo™ assay. BDQ was used as positive control at 25x its MIC. Every condition was tested in triplicate, and the experiment was repeated once.

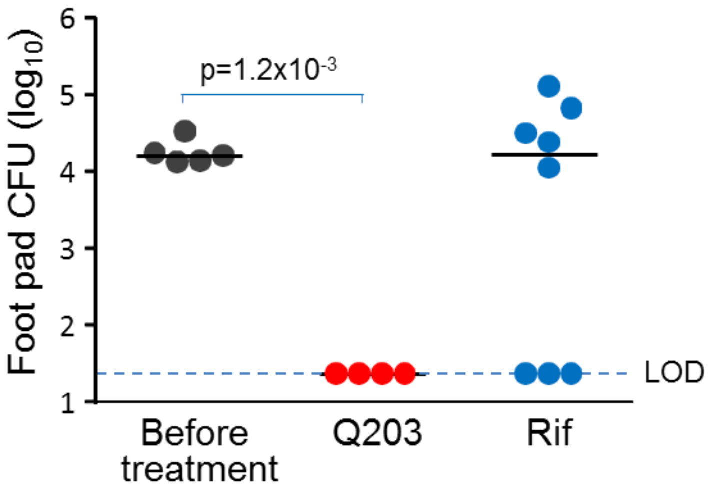

Supplementary Figure 8. Intermittent dosing of Q203 is effective at reducing bacterial load in mice infected with *M. ulcerans* S1013. From the results reported on Figure 4b, bacterial load in infected foot pad was determined by CFU determination 6 weeks after the end of drug treatment (week 15 post-infection). The intended group size was five mice before treatment, and 4 mice for each group after treatment. However, all the animals from the rifampicin (rif)-treated group had to be euthanized at week 15 due to unfavorable disease progression in 5/8 animals. No animals were excluded for the analysis. No bacteria were detected in any of the four animals treated with Q203. Black bars represent the median values. Student's t-test was used for statistical analysis. Dotted blue line: limit of detection (LOD).

Supplementary Table 1: Structure activity relationship of the IPA series against the classical *M. ulcerans* African strain S1013. Compounds are ranked according to their potency.

MIC µg/ml	N (cpds)
< 0.001	1
0.001 - 0.01	6
0.01 - 0.1	11
0.1 - 1	22
1.0 - 10.0	14
> 10	31
total	85

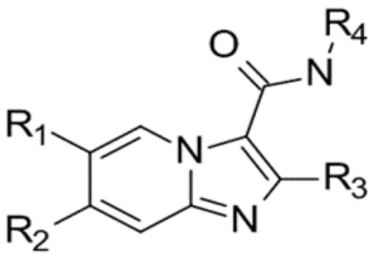

Number	R1	R2	R3	R4	MIC range
Q203 (1)	Cl	H	Et	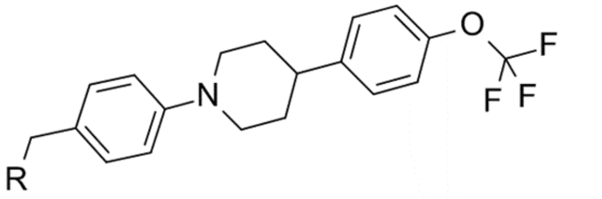	
2	Me	H	Me	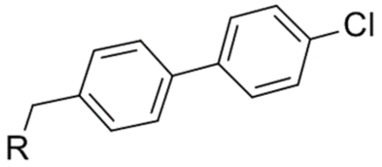	
3	H	Me	Me	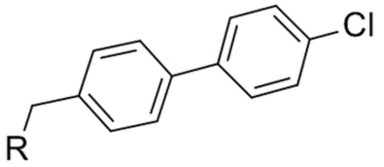	
4	Me	H	Me	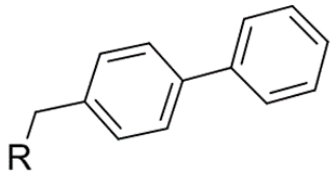	
5	Cl	H	Et	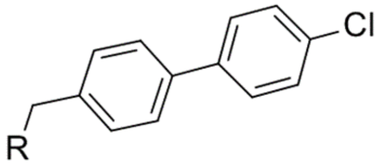	
6	Cl	H	Et	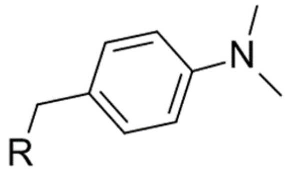	

7	Cl	H	Me	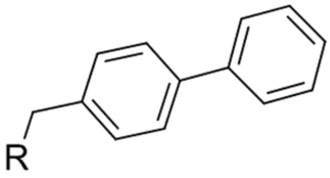	
8	Br	H	Me	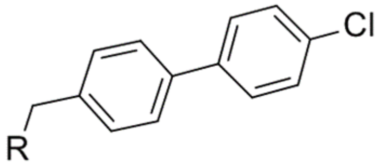	
9	Cl	H	Et	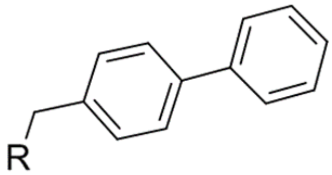	
10	Me	H	Me	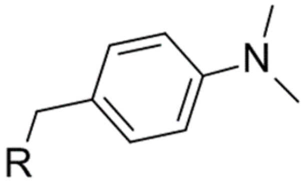	
11	Br	H	Me	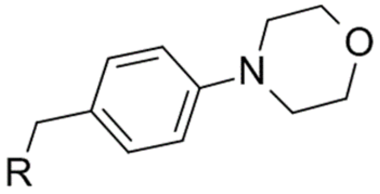	
12	Cl	H	Et	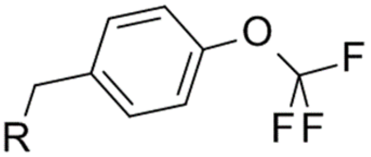	
13	Me	H	Me	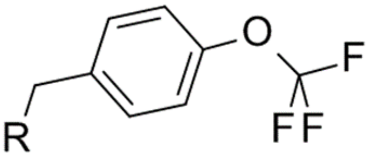	
14	Br	H	Me	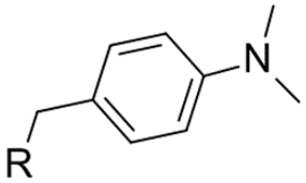	
15	H	H	Cl	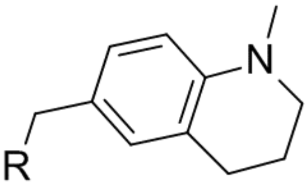	

16	H	H	Cl	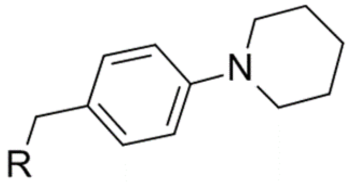	
17	Cl	H	Me	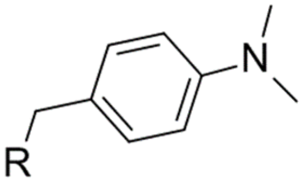	
18	H	H	Cl	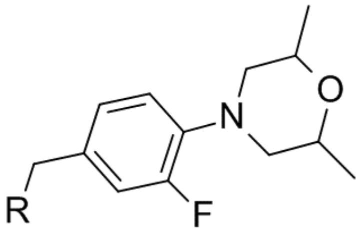	
19	Cl	H	Me	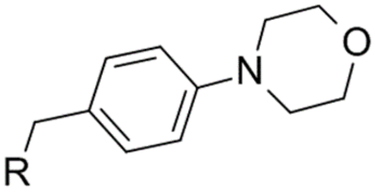	
20	H	H	Cl	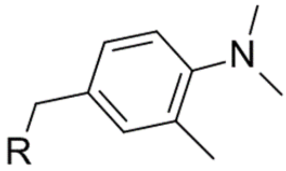	
21	H	H	Cl	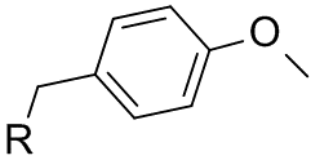	
22	H	H	Me	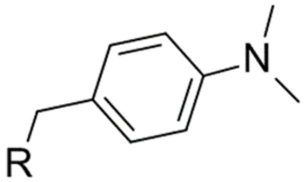	
23	Cl	H	Me	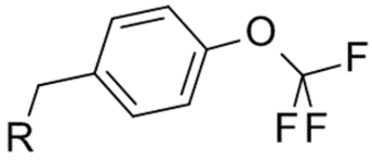	
24	H	Me	Me	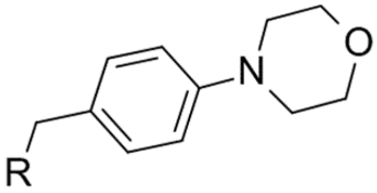	

25	H	H	Me	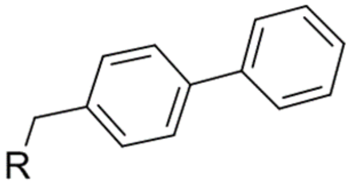	
26	H	H	Cl	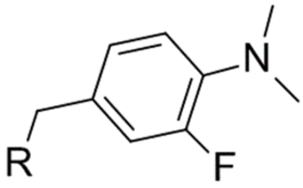	
27	H	H	Me	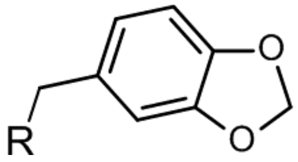	
28	H	H	Cl	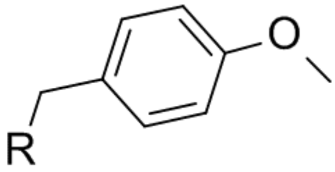	
29	H	H	Cl	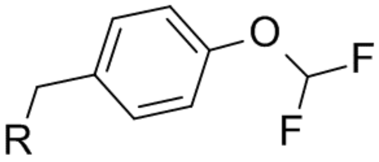	
30	Cl	H	Et	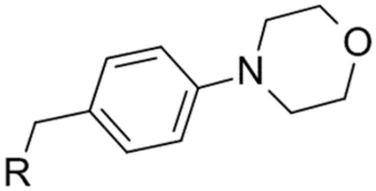	
31	H	H	Cl	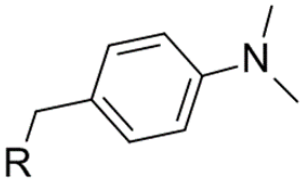	
32	H	Me	Me	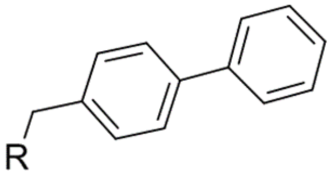	
33	H	H	Me	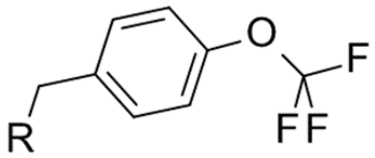	

34	H	H	Cl	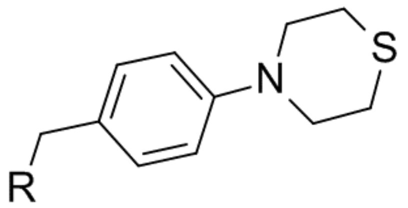	
35	Br	H	Me	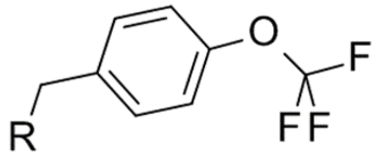	
36	H	H	Me	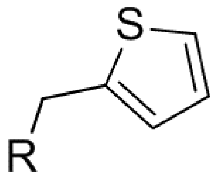	
37	H	H	Cl	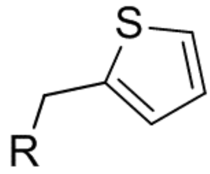	
38	H	Me	Me	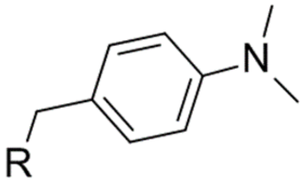	
39	H	Me	Me	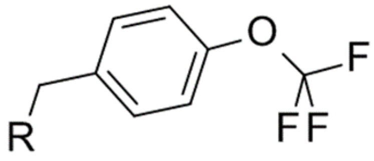	
40	H	H	Cl	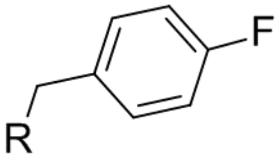	
41	H	H	Me	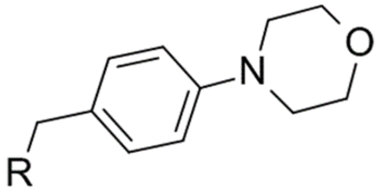	
42	H	H	Cl	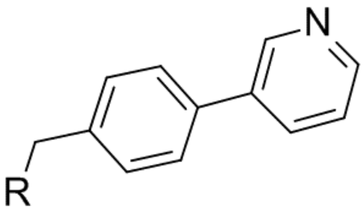	

43	H	H	Cl	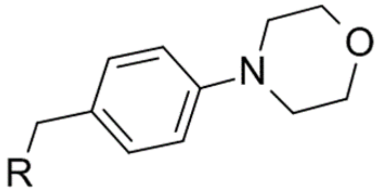	
44	H	H	Cl	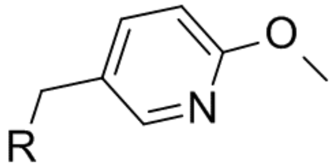	
45	H	H	Cl	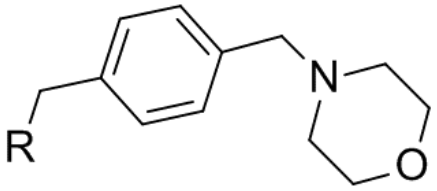	
46	Me	H	H	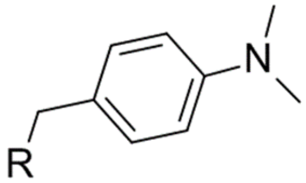	
47	Me	H	H	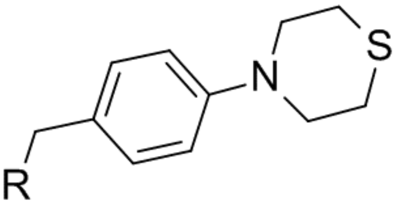	
48	Me	H	Me	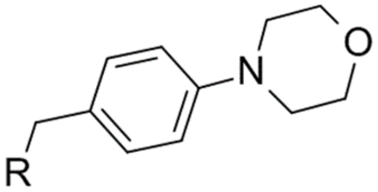	
49	H	H	Me	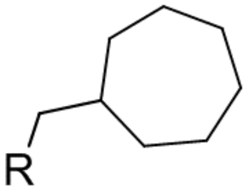	
50	H	H	Cl	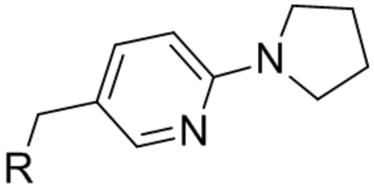	

51	H	H	Cl	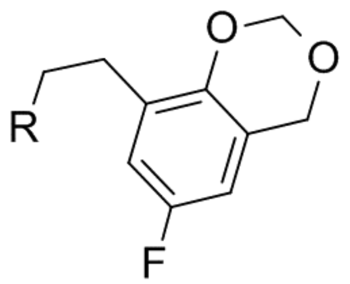	
52	H	H	Cl	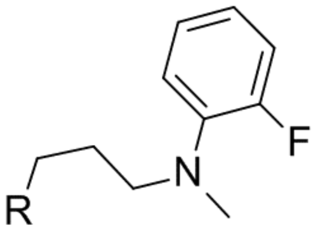	
53	H	H	Cl	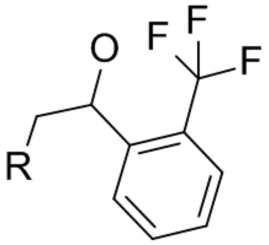	
54	H	H	Cl	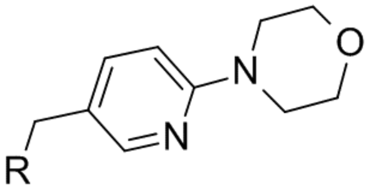	
55	Me	H	H	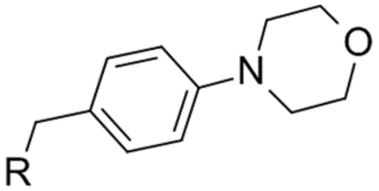	
56	H	H	Me	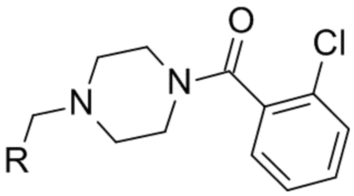	
57	H	H	Cl	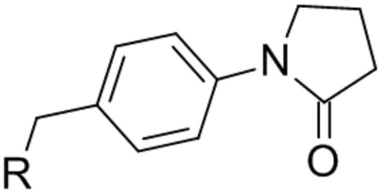	
58	H	H	Cl	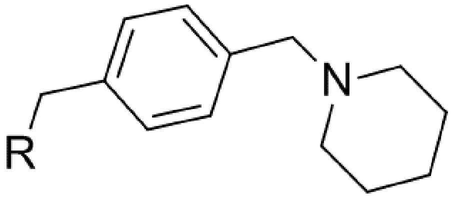	

59	H	H	Cl	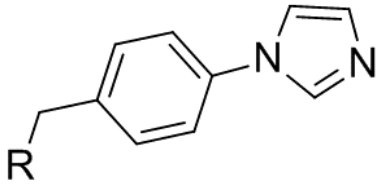	
60	H	H	Cl	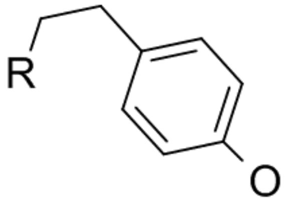	
61	H	H	Cl	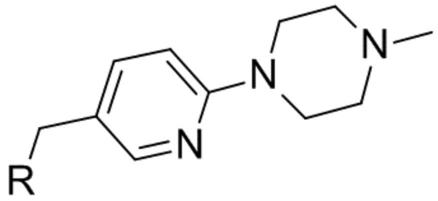	
62	H	Me	Me	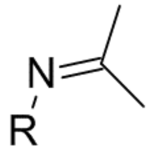	
63	H	H	Me	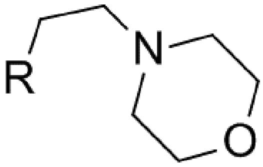	
64	H	H	Me	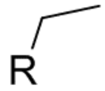	
65	H	H	p-Me-Ph	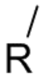	
66	H	H	Cl	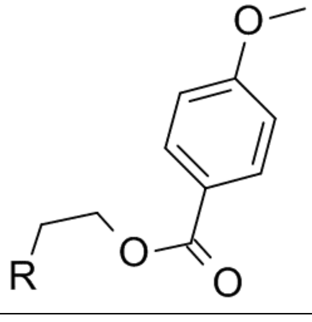	

67	H	H	Cl	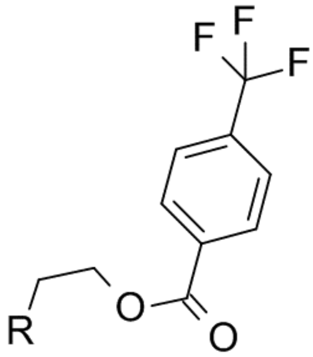	
68	H	H	Cl	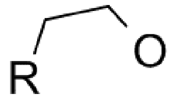	
69	Cl	H	Me	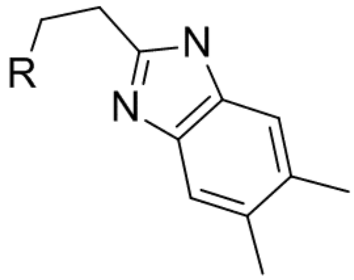	
70	Me	H	m-Me-Ph	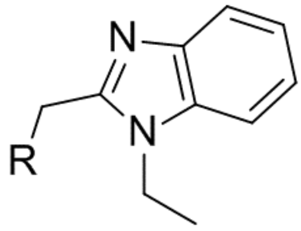	
71	H	H	Cl	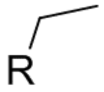	
72	H	H	Cl	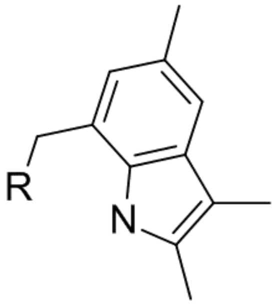	

73	H	H	Cl	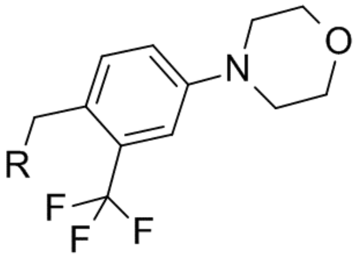	
74	H	H	Cl	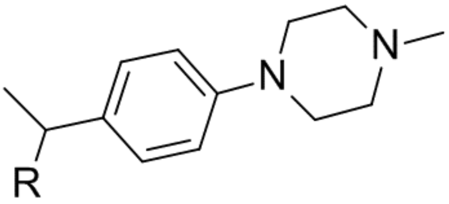	
75	H	Me	Me	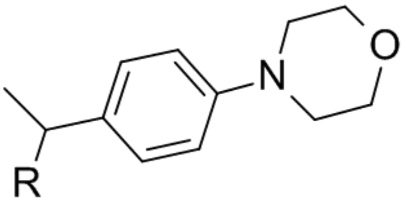	
76	H	H	Cl	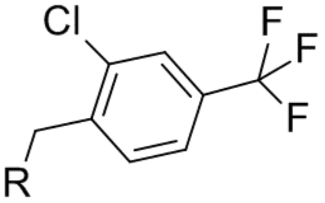	
77	H	H	Cl	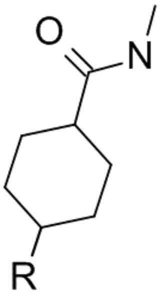	
78	H	Me	H	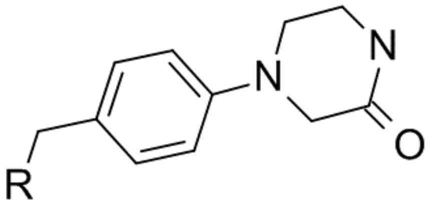	
79	H	H	Me	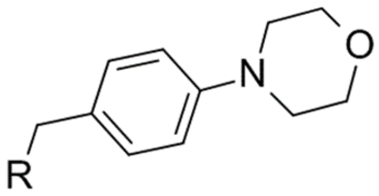	

80	H	H	Me	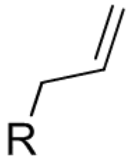	
81	H	H	Me	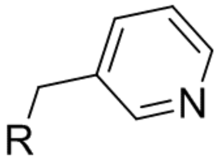	
82	H	Me	Me	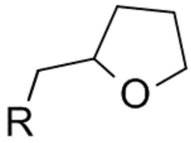	
83	H	H	Me	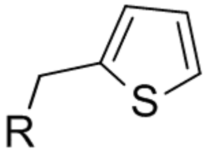	
84	H	Me	Me	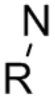	
85	H	H	Me	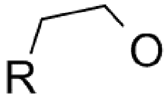	

Supplementary Table 2. Mapping of the mutations in *qcrB* from spontaneous resistant clones of *M. ulcerans* ST1013 selected on Q203. Spontaneous resistant clones were selected on 7H10-OADC agar plates supplemented with 10 nM of Q203. Fold resistance shift to Q203, ND-11176, and bedaquiline (BDQ) was determined by calculating the ration between the MIC_{50} values of the spontaneous resistant clones and the MIC_{50} values of the parental strain. n.d.: not determined.

Clones #	*QcrB* SNP	QcrB subsitution	Fold resistance to :		
			Q203	ND-11176	BDQ
1, 7, 8, 9, 11	A967G	T323A	230.5	>270	0.9
2, 3, 5, 6, 10, 13, 14	A1054G	M352V	8.9	6.5	0.5
4	T1055C	M352T	n.d.	n.d.	n.d.
12	G966T	W322C	n.d.	n.d.	n.d.

Supplementary Table 3. PK/PD parameters of Q203 in mice. Pharmacokinetic parameters were determined after administration of a single oral dose of 2 mg/kg.

	**Q203**	**Q203**
Dose (mg/kg)	**2.00**	**0.5***
C_{max} (ng/mL)	1,709.9	427*
T_{max} (h)	4.00	4.0
$AUC_{0\text{-}last}$ (ng·h/mL)	20,693	5,173*
AUC/MIC	37,150	9,287
Cmax/MIC	3,069.8	766
T>MIC (%)	100	100

*Extrapolated values assuming plasma levels are proportional to the dose.

Cmax: maximum plasma drug concentration; AUC: area under the curve; AUC/MIC: ratio between plasma exposure and MIC (MIC value rounded at 1 nM, or 0.557 ng/ml); T>MIC: % of the time during which plasma levels exceed the MIC.
