# Supplementary material for: Targeting the Mycobacterium ulcerans cytochrome bc1:aa3 for the treatment of Buruli ulcer
Source: Nat Commun. 2018 Dec 18;9:5370. doi: 10.1038/s41467-018-07804-8 (PMC6299076; doi:10.1038/s41467-018-07804-8)
Supplement: Supplementary file 2 — Reporting Summary [file 41467_2018_7804_MOESM2_ESM.pdf]

nature research

Corresponding author(s): Kevin Pethe

Reporting Summary

Nature Research wishes to improve the reproducibility of the work that we publish. This form provides structure for consistency and transparency in reporting. For further information on Nature Research policies, see Authors & Referees and the Editorial Policy Checklist.

Statistical parameters

When statistical analyses are reported, confirm that the following items are present in the relevant location (e.g. figure legend, table legend, main text, or Methods section).

n/a	Confirmed	
☐	☒	The exact sample size (n) for each experimental group/condition, given as a discrete number and unit of measurement
☐	☒	An indication of whether measurements were taken from distinct samples or whether the same sample was measured repeatedly
☐	☒	The statistical test(s) used AND whether they are one- or two-sided<br>*Only common tests should be described solely by name; describe more complex techniques in the Methods section.*
☒	☐	A description of all covariates tested
☒	☐	A description of any assumptions or corrections, such as tests of normality and adjustment for multiple comparisons
☒	☐	A full description of the statistics including central tendency (e.g. means) or other basic estimates (e.g. regression coefficient) AND variation (e.g. standard deviation) or associated estimates of uncertainty (e.g. confidence intervals)
☒	☐	For null hypothesis testing, the test statistic (e.g. F, t, r) with confidence intervals, effect sizes, degrees of freedom and P value noted<br>*Give P values as exact values whenever suitable.*
☒	☐	For Bayesian analysis, information on the choice of priors and Markov chain Monte Carlo settings
☒	☐	For hierarchical and complex designs, identification of the appropriate level for tests and full reporting of outcomes
☒	☐	Estimates of effect sizes (e.g. Cohen's d, Pearson's r), indicating how they were calculated
☐	☒	Clearly defined error bars<br>*State explicitly what error bars represent (e.g. SD, SE, CI)*

Our web collection on statistics for biologists may be useful.

Software and code

Policy information about availability of computer code

Data collection	NA
Data analysis	NA

For manuscripts utilizing custom algorithms or software that are central to the research but not yet described in published literature, software must be made available to editors/reviewers upon request. We strongly encourage code deposition in a community repository (e.g. GitHub). See the Nature Research guidelines for submitting code & software for further information.

Data

Policy information about availability of data

All manuscripts must include a data availability statement. This statement should provide the following information, where applicable:

- Accession codes, unique identifiers, or web links for publicly available datasets
- A list of figures that have associated raw data
- A description of any restrictions on data availability

No restriction to data availability

Field-specific reporting

Please select the best fit for your research. If you are not sure, read the appropriate sections before making your selection.

☒ Life sciences ☐ Behavioural & social sciences ☐ Ecological, evolutionary & environmental sciences

For a reference copy of the document with all sections, see nature.com/authors/policies/ReportingSummary-flat.pdf

Life sciences study design

All studies must disclose on these points even when the disclosure is negative.

Sample size	Triplicate is standard practice in the field that gives a good idea of data distribution, while keeping number technically manageable. For the animal experiment, 4 to 10 animal per conditions were used to captured difference in response to treatment. For CFU determination, 10 animals per conditions and time points were used because samples get frequently contaminated and lost for analysis. This is because Mycobacterium ulcerans is growing extremely slow and any bacteria or fungi contamination will impact CFU determination ins some samples. Four animals per time point and conditions were used for the histopathology studies.We did not use a statistical method to predetermine animal ample size. The sample size was constrained by ethical use of animals and expense of the study.
Data exclusions	No data were excluded except in vivo samples that could be counted due to heavy bacterial and/or fungal contaminations because M. ulcerans bacterial load could not be determined.
Replication	The experiments related to antibacterial activity and mode of action studies were repeated at least once and were confirmed. Selection of escape mutants to Q203 was performed once. Animals experiments were performed independently in two different laboratories in Switzerland and France.
Randomization	For the animal study, mice were randomly assigned to different groups. prior treatment.
Blinding	No blinding was used

Reporting for specific materials, systems and methods

Materials & experimental systems

n/a	Involved in the study	
☒	☐	Unique biological materials
☒	☐	Antibodies
☒	☐	Eukaryotic cell lines
☒	☐	Palaeontology
☐	☒	Animals and other organisms
☒	☐	Human research participants

Methods

n/a	Involved in the study	
☒	☐	ChIP-seq
☒	☐	Flow cytometry
☒	☐	MRI-based neuroimaging

Animals and other organisms

Policy information about studies involving animals; ARRIVE guidelines recommended for reporting animal research

Laboratory animals	Female BALB/c mice were used for the challenge studies. Studies were approved by the Institutional Animal Care and Use Committee (IACUC) listed in the methods section of the manuscript
Wild animals	The study did not involve wild animals
Field-collected samples	The study did not involve sample collected from the field
